# A partially hydrolyzed 100% whey formula and the risk of eczema and any allergy: an updated meta-analysis

**DOI:** 10.1186/s40413-017-0158-z

**Published:** 2017-07-26

**Authors:** Hania Szajewska, Andrea Horvath

**Affiliations:** Department of Paediatrics, The Medical University of Warsaw, Żwirki i Wigury 63A, 02-091 Warsaw, Poland

**Keywords:** RCT, Children, Prevention, Allergy, Protein hydrolysates

## Abstract

**Background:**

Recently, the role of using hydrolyzed formula for the prevention of allergic disease has been questioned. However, not all hydrolyzed formulas are equal. The efficacy of each hydrolyzed formula should be established separately. We updated evidence on the effectiveness of using partially hydrolyzed 100% whey formula (pHF), manufactured by a single manufacturer, for reducing the risk of eczema and allergy in healthy infants at high risk for allergy.

**Methods:**

The Cochrane Library, MEDLINE, and EMBASE databases were searched in June 2016 for randomized and quasi-randomized controlled trials (RCTs); additional data were obtained from reviewed articles and the authors of included trials.

**Results:**

Thirteen publications reporting on eight RCTs were included. Use of pHF compared to cow’s milk formula reduced the risk of eczema and all allergic diseases among children at high risk for allergy. Both intention-to-treat analyses and per-protocol analyses showed that the reduction was statistically significant at some, albeit not all, time points.

**Conclusions:**

There is evidence to consider use of pHF as an option for reducing the risk of any allergic diseases, particularly eczema. However, the certainty of the evidence is low. One characteristic that makes our meta-analysis distinct from other reviews is that it focuses exclusively on only one type of pHF.

**Electronic supplementary material:**

The online version of this article (doi:10.1186/s40413-017-0158-z) contains supplementary material, which is available to authorized users.

## Background

Most guidelines and experts recommend that infants with a documented hereditary risk of allergy (i.e., an affected parent and/or sibling) who cannot be breastfed exclusively should receive a formula with confirmed reduced allergenicity, i.e., a partially or extensively hydrolyzed formula, as a means of preventing allergic reactions, primarily atopic dermatitis [1–4]. However, different opinions exist. Recently, the Australasian Society of Clinical Immunology and Allergy concluded that there is ‘*no consistent convincing evidence to support a protective role for partially hydrolyzed formulas (usually labelled ‘HA’ or hypoallergenic) or extensively hydrolyzed formulas for the prevention of eczema, food allergy, asthma or allergic rhinitis in infants and children’* [5]. The latter position was based on results from a 2016 meta-analysis by Boyle et al. [6], which questioned the role of hydrolyzed formula for the prevention of allergic disease. This meta-analysis found no consistent evidence that use of partially or extensively hydrolyzed formulas reduces the risk of allergic outcomes in infants at high pre-existing risk of these outcomes. While the authors of this review evaluated separately partially and extensively formulas, various types of hydrolyzed formulas in each category were combined. However, not all hydrolyzed formulas are equal. The efficacy and safety should be established for each hydrolyzed formula, as factors such as the protein source, hydrolysis method, and degree of hydrolysis that often depend on the manufacturer contribute to differences among hydrolysates.

In 2010, given the latter consideration, we reviewed data on the efficacy of using only one type of hydrolyzed formula, i.e., a partially hydrolyzed 100% whey formula [7]. Our meta-analysis showed that this formula compared to cow’s milk formula (CMF) reduced the risk of all allergic diseases, particularly atopic dermatitis/eczema, at some time points, albeit not all, among children at high risk for allergy. Similar conclusions were reached by the authors of another meta-analysis [8].

We maintain our position that each hydrolyzed formula, from each manufacturer, should be evaluated separately. Considering the uncertainty regarding the actual efficacy of a particular hydrolyzed formula raised by Boyle et al. [6] and considering that new data have been published since our meta-analysis [7], our aim was to systematically update our 2010 meta-analysis. Here, we report results of an updated meta-analysis on the efficacy of a partially hydrolyzed 100% whey formula manufactured by one company (*Nestlé*) (hereafter, this formula is referred to as pHF) compared with a cow’s milk-based infant formula in reducing the risk of eczema and allergy in healthy infants at high risk for allergy.

## Methods

The guidelines from the Cochrane Collaboration for undertaking and reporting the results of this systematic review and meta-analysis, as well as the PRISMA guidelines, were followed [9, 10]. For working protocol, see Additional file 1.

### Criteria for considering studies for this review

#### Type of studies

All relevant randomized controlled trials (RCTs) were eligible for inclusion. To supplement existing randomized trial evidence, quasi-randomized controlled trials were also reviewed. The latter are studies in which the participants are allocated to different interventions using methods that are not random. For example, allocation may be based on the person’s date of birth, the person’s medical record number, or the day of the week or month of the year.

#### Type of participants

Participants had to be healthy term infants at high risk of developing allergy, as assessed by a family history (the presence of allergy in at least one parent and/or sibling) and/or other markers (as determined by the study investigators).

#### Type of interventions

We included trials that compared use of the pHF compared with a regular CMF. If other experimental arms were available, they were not considered.

#### Type of outcomes

We focused on two outcomes that are currently under discussion. The first outcome was eczema. Our decision to focus primarily on eczema was driven by results of previous trials and systematic reviews showing that, if there is an effect of hydrolyzed formulas, it is the reduction of the risk of eczema. For a more complete picture, ‘all allergic disease’ (as defined by the authors of original publications) was added as an outcome, as it was also considered in our previous review. We report outcomes at time intervals reported by the authors of the original publications (i.e., at 1 y, 2 y, 3 y, 5–6 y, 6–7 y, 10 y, and 15 y).

### Search methods for identification of studies

For details on electronic searches, data collection and analysis, and data extraction and management, see Additional file 2: Data S1.

### Assessment of risk of bias in included studies

The Cochrane Collaboration’s tool for assessing risk of bias was used to establish the risk of bias (see also Additional file 2: Data S1) [11].

### Measures of treatment effect

The data were entered into Review Manager (RevMan) [Computer program; Version 5.3. Copenhagen: The Nordic Cochrane Centre, The Cochrane Collaboration, 2014] for analysis. The results for individual studies and pooled statistics are reported as the risk ratio (RR) between the experimental and control groups with 95% confidence intervals (95% CI).

### Dealing with missing data

We assessed pooled data using intention-to-treat analysis, i.e., an analysis in which data are analyzed for every participant for whom the outcome was obtained (also known as available case analysis), rather than intention-to-treat analysis with imputation [12]. We also report results of per-protocol analysis. The latter included all participants who adhered adequately to the assigned regimen.

### Assessment of heterogeneity

Heterogeneity was quantified by χ^2^ and *I* ^2^. A value for *I* ^2^ of 0% indicates no observed heterogeneity, and larger values show increasing heterogeneity. All analyses were based on the random effects model.

### Assessment of reporting biases

To test for publication bias, a test for asymmetry of the funnel plot, as proposed by Egger et al. [13], was planned; however, sufficient (≥10) eligible trials were not available for any given outcome.

### Data synthesis

The data were analyzed using Review Manager (RevMan) [Computer program; Version 5.3. Copenhagen: The Nordic Cochrane Centre, The Cochrane Collaboration, 2014]. The numbers needed to treat (NNT) were derived from the pooled RR using StatsDirect Statistical Software (version 3.0.171 [08.04.2016]).

### Quality of evidence

For assessing the quality of evidence (also known as certainty in the evidence or confidence in the effect estimates) for outcomes reported in the included studies, we chose to use the GRADE methodology and GRADEProfiler software (version 3.6, 2011). The GRADE system offers four categories of the quality of the evidence (i.e., high, moderate, low, and very low) [14].

## Results

### Description of studies

Additional file 2: Table S1 summarizes characteristics of all of 13 publications reporting on eight RCTs [15–27]. For a flow diagram documenting the identification process for eligible trials, as well as the characteristics of the excluded trials, with reasons for exclusion, see Additional file 2: Table S2 and Fig. S1. Compared with our 2010 meta-analysis, three new publications published subsequently were included [17, 25, 26].

Among the three new publications, there were two publications reporting a 10-year [25] and 15-year [26] follow-up of the German Infant Nutritional Intervention study (GINI study). This was the largest, included, double-blind RCT, which involved 2252 infants, among them 557 infants who received pHF and 556 who received CMF. The third publication reported findings of the Melbourne Atopy Cohort Study (MACS) [17]. This was a single-blind RCT involving 620 infants designed to compare the effects of use of several types of infant formula at weaning on the risk of allergic disease. The participants were randomized to receive, at partial or full cessation of breastfeeding, one of three infant formulas: cow’s milk formula (CMF, *n* = 206), soy formula (*n* = 208), or pHF (*n* = 206). Study formulas were offered until the end of the first year of life.

Overall, the included trials involved 2057 participants (1012 in the pHF groups and 1045 in the control groups). The sample size ranged from 33 to 1113. Only 2 RCTs [17, 22] provided sample size calculations.

All included studies were published in peer-reviewed journals. A number of trials described the same population at different time points [20–24].

All included trials were performed in industrialized countries. All of the studies were carried out in infants and children at high risk of allergy. The duration of the intervention varied from 3 to 12 months. In four study populations, the experimental formulas were used exclusively from birth [15, 16, 20, 21, 27]. In the remaining trials, the study formula was recommended in addition to breastfeeding [17, 21]. In seven study populations, there was no co-intervention [15, 19, 20, 22, 27]. In the remaining trials, additional co-interventions were recommended. The children were followed up from 4 months to 15 years.

One trial (the GINI study) was independently funded by public institutions, and then by non-industry funding until the 10-year follow-up [22–24]. Later on, industrial support, including that from a pHF manufacturer, was obtained [25, 26]. Five trials were funded by the pHF manufacturer [15, 17, 19–21]; in two trials, the source of funding was not specified [16, 18], and in one trial, the source of funding was unclear [27] (Additional file 2: Table S1).

### Risk of bias in included studies

The included studies are described with respect to their risk of bias across the included RCTs in Additional file 2: Fig. S2. The risk of bias for each included trial showed that with the exception of one RCT by von Berg et al. reporting at 1 year [22] and at 3 years of age [23], all included trials had a number of methodological limitations (Additional file 2: Fig. S3).

The GRADE assessment for outcomes related to use of pHF and risk of eczema is presented in Additional file 2: Tables S3. Using the GRADE, the overall quality of evidence for all assessed outcomes was rated as moderate to very low.

### Effects of interventions

#### pHF versus CMF

##### Eczema

**Fig. 1:**
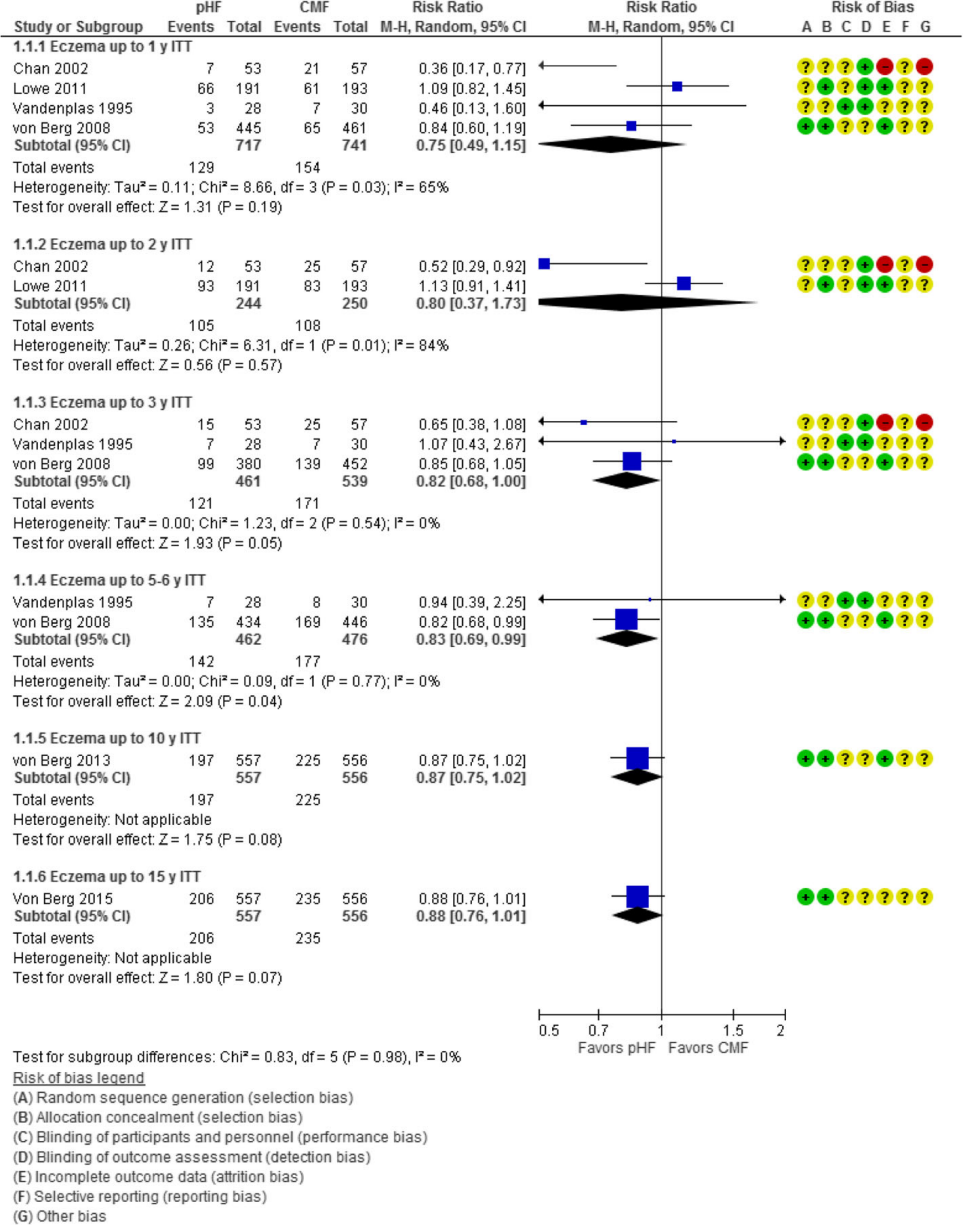
Partially hydrolyzed formula (pHF) vs. cow’s milk formula (CMF). Eczema (cumulative incidence, ITT analysis)

Six trials [15, 17, 21, 24–26] reported the effect of use of pHF on the *cumulative incidence* of eczema (Fig. 1)*.* The pooled results of data within a given period (0 to 1 year, 0 to 2 years, 0 to 3 years, 0 to 5–6 years, 0 to 10 years, and 0 to 15 years) showed a reduction in the risk of eczema in favor of pHF compared with CMF, which in the intention-to-treat (ITT) analyses was statistically significant at the time intervals of 0 to 3 years (3 RCTs, *n* = 1000, RR 0.82, 95% CI 0.68 to 1.00) and 0 to 5–6 years (2 RCTs, *n* = 938, RR 0.83, 95% CI 0.69 to 0.99). At two time intervals (0 to 10 years and 0 to 15 years), the results were of borderline statistical significance in favor of pHF.

**Fig. 2:**
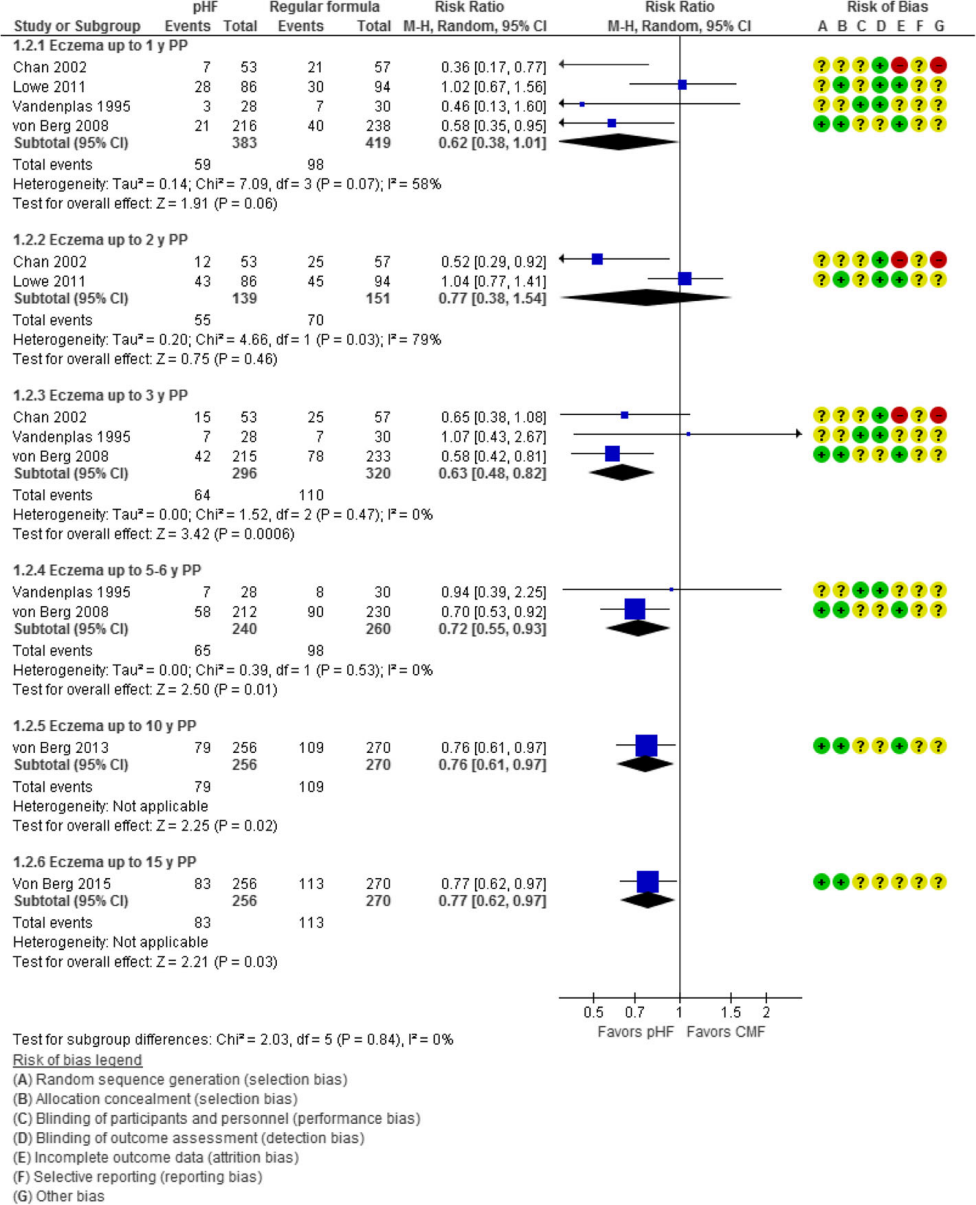
Partially hydrolyzed formula (pHF) vs. cow’s milk formula (CMF). Eczema (cumulative incidence, PP analysis)

Per-protocol analyses showed a reduction in the risk of eczema in favor of pHF compared with CMF, which was statistically significant at 0 to 3 y (3 RCTs, *n* = 616, RR 0.63, 95% CI 0.48 to 0.82) and at 0 to 5–6 y (2 RCTs, *n* = 500, RR 0.72, 95% CI 0.55 to 0.93) (Fig. 2).

**Fig. 3:**
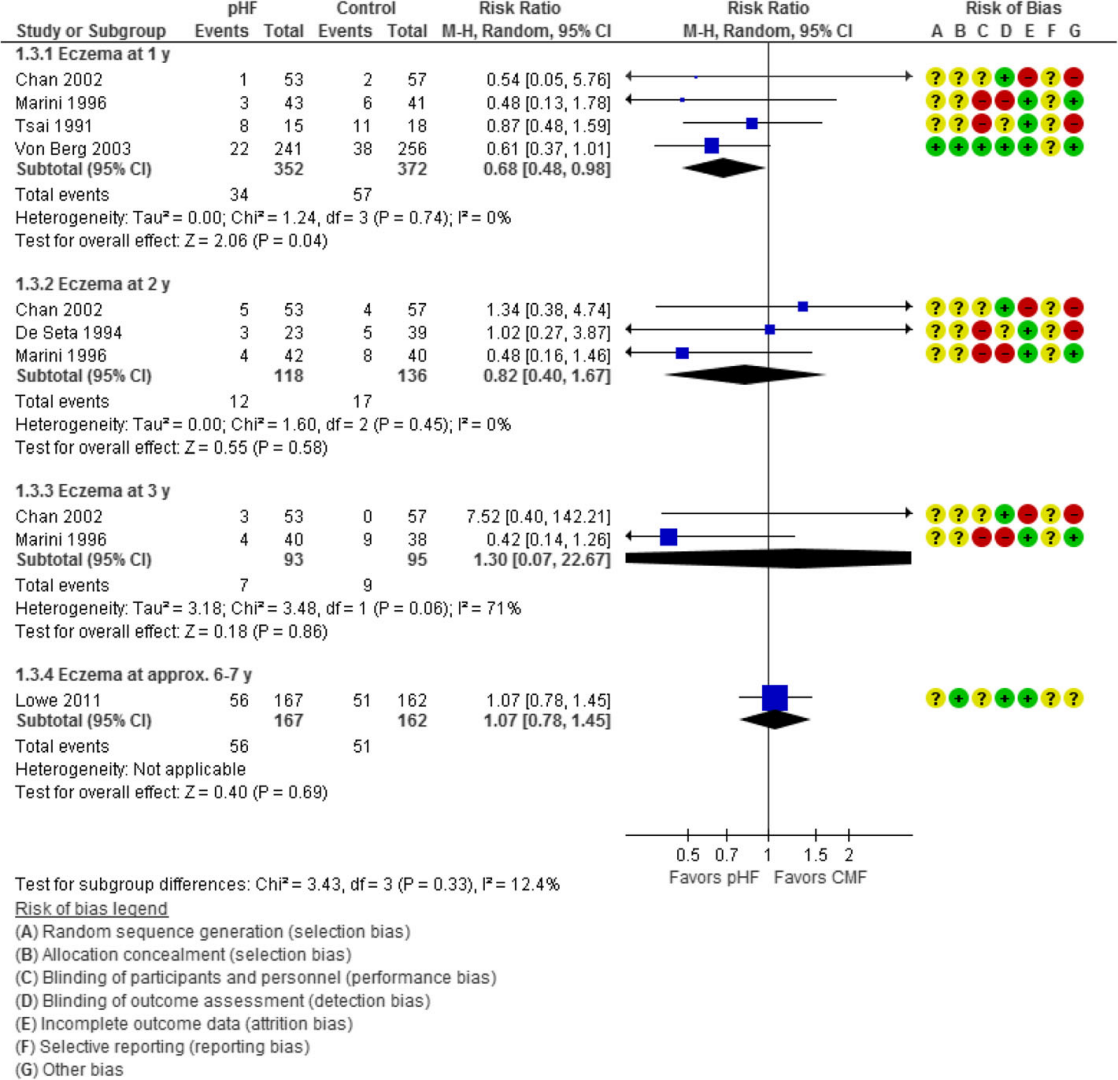
Partially hydrolyzed formula (pHF) vs. cow’s milk formula (CMF). Eczema (incidence)

Six trials [15–17, 22] reported the effect of use of pHF compared with CMF on the incidence of eczema at certain time points. At 1 y, use of pHF compared with CMF reduced the risk of eczema (4 RCTs, *n* = 724, RR 0.68, 95% CI 0.48 to 0.98). No significant heterogeneity was found (Chi2 = 1.24, *P* = 0.74, *I2 =* 0%). For every 18 patients receiving pHF, one fewer would develop eczema at 1 year (NNT 18, 95% CI 10 to 119). However, there was no significant difference in the incidence of eczema between the pHF and CMF groups at 2 y, 3 y, or at 6–7 years. Heterogeneity was found at 3 years only (Chi2 = 3.48, *P* = 0.06, *I2 =* 71%) (Fig. 3).

##### All allergic diseases

**Fig. 4:**
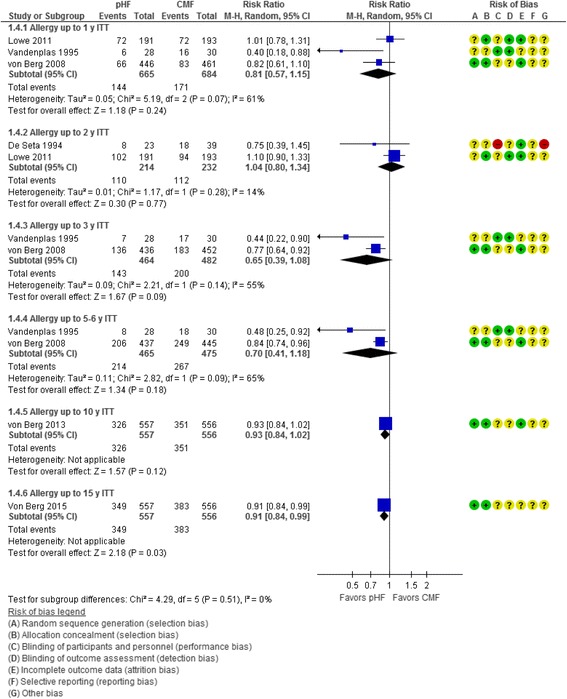
Partially hydrolyzed formula (pHF) vs. cow’s milk formula (CMF). All allergic diseases (cumulative incidence, ITT analysis)

**Fig. 5:**
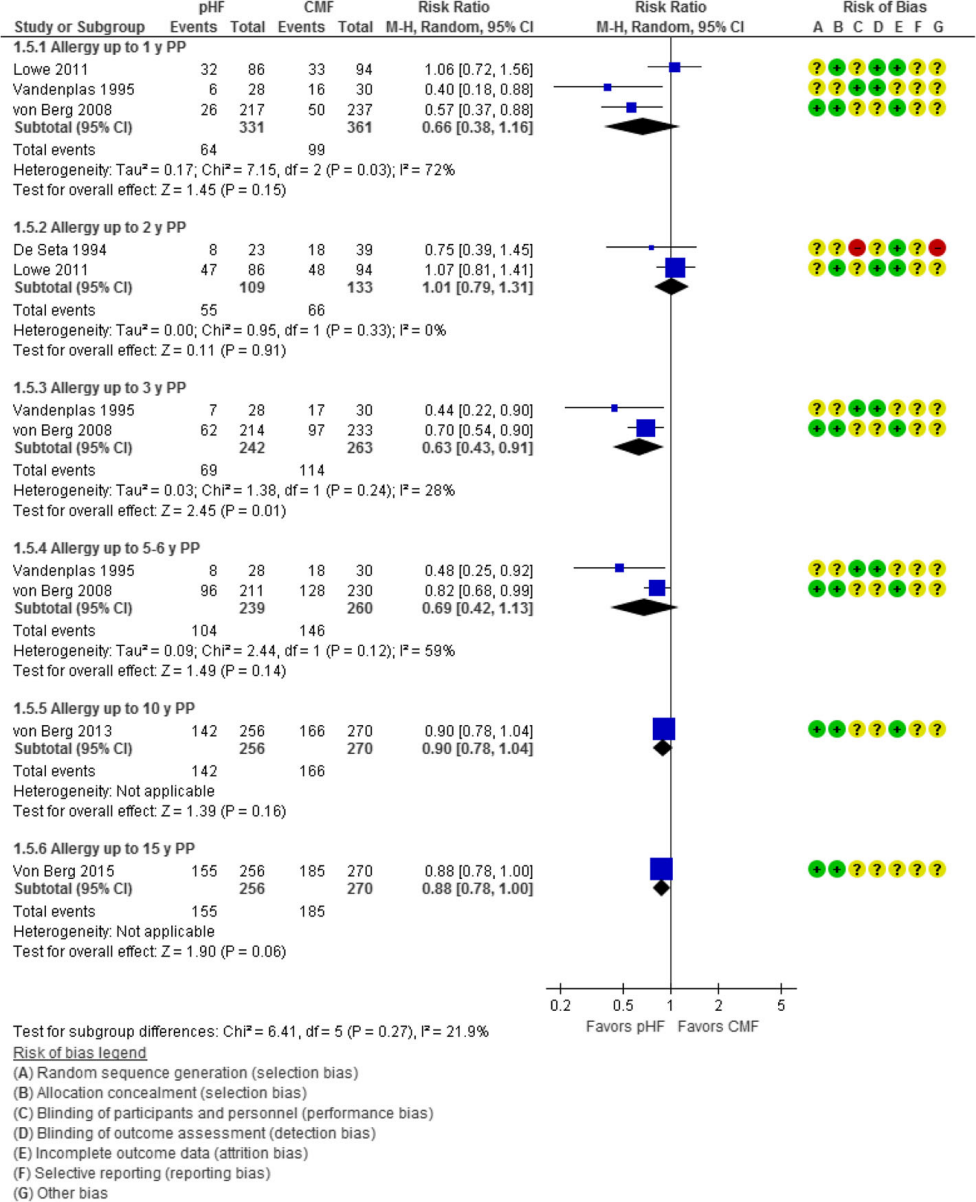
Partially hydrolyzed formula (pHF) vs. cow’s milk formula (CMF). All allergic diseases (cumulative incidence, PP analysis)

Six publications reported data on the incidence of *all allergic diseases* [16, 17, 21, 24–26]. Meta-analyses of the data showed a reduced risk of all allergic diseases in favor of pHF compared with CMF; however, the results were only statistically significant in the ITT analyses at 0 to 15 years (1 RCT, *n* = 1113, RR 0.91, 95% CI 0.84 to 0.99)(Fig. 4). In the per-protocol analyses, the difference was only statistically significant at 0 to 3 y (2 RCTs, *n* = 505, RR 0.63, 95% CI 0.43 to 0.91) and at 0 to 15 y (1 RCT, *n* = 526, RR 0.88, 95% CI 0.78 to 1.00)(Fig. 5).

**Fig. 6:**
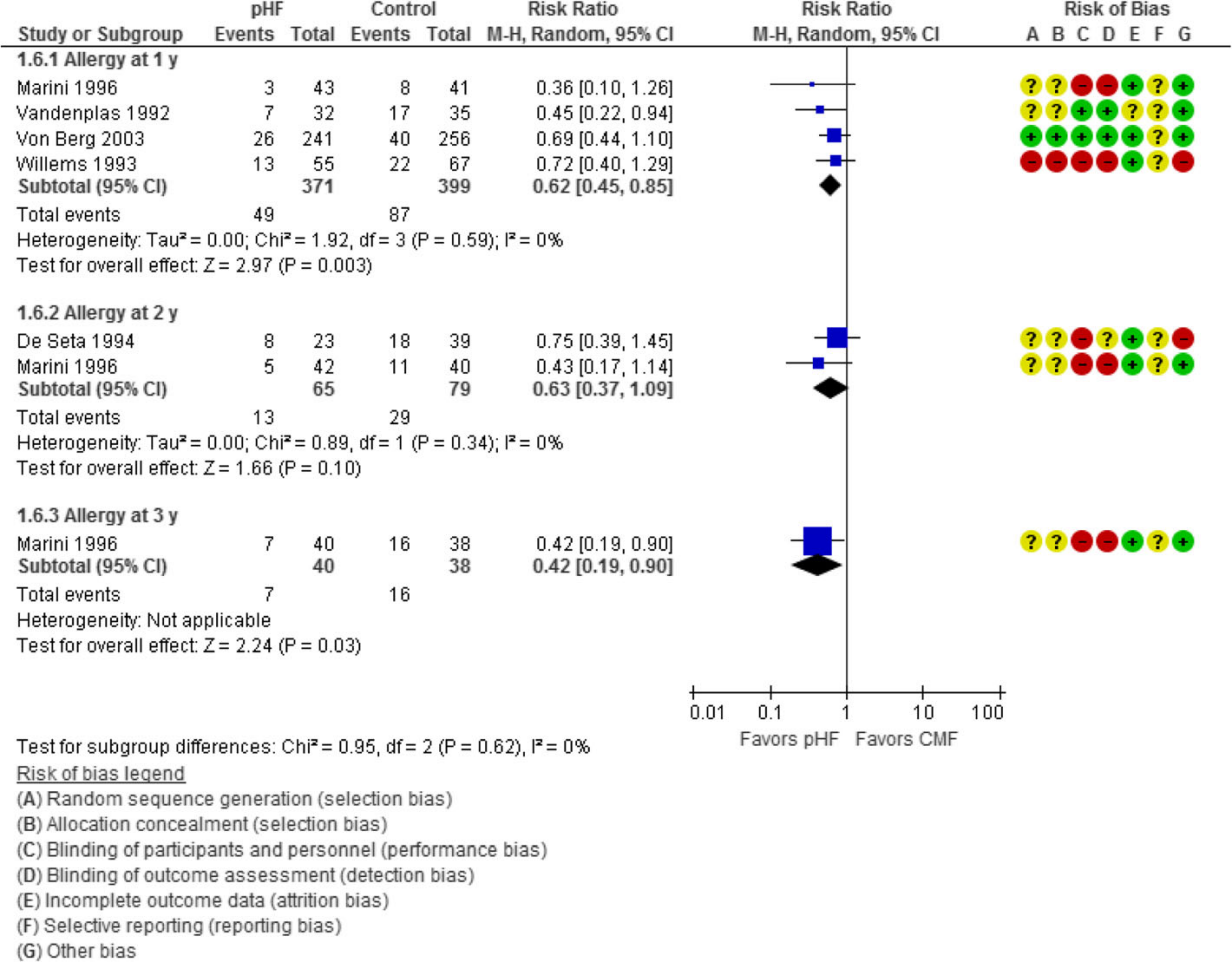
Partially hydrolyzed formula (pHF) vs. cow’s milk formula (CMF). All allergic diseases (incidence).

Five trials [16, 18, 20, 22, 27] reported the effects of use of pHF compared with CMF on the incidence of all allergic diseases at various time points. At 1 y, use of pHF compared with CMF reduced the risk of all allergic diseases (4 RCTs, *n* = 770, RR 0.62, 95% CI 0.45 to 0.85). No significant heterogeneity was found (Chi2 = 1.92, *P* = 0.59, *I2 =* 0%). For every 12 patients receiving pHF, one fewer would develop allergy at 1 year (NNT 12, 95% CI 8 to 31). There was also a significant difference between groups at 3 y in favor of pHF (1 RCT, *n* = 78, RR 0.42, 95% CI 0.19 to 0.90)(Fig. 6).

## Discussion

### Main findings

This updated meta-analysis of RCTs and quasi-RCTs confirmed that use of pHF compared to CMF reduced the risks of eczema and all allergic diseases among children at high risk for allergy. Both ITT analyses and per-protocol analyses showed that the reduction was statistically significant only at some, albeit not all, time points. Some results were of borderline statistical significance in favor of pHF, i.e., the upper limit of the 95% CI for RR was 1.01 or 1.02. As the 95% CI for RR included 1, there was no significant difference between treatments in these cases. However, we cannot conclude that these results are not clinically important. For example, 95% CIs ranging from 0.75 to 1.02 included both RRs of clinical importance and RRs of no importance. It is possible that the sample size was too small to allow confidence about where the true result lies.

Interestingly, two studies that contributed the most to the pooled results showed opposite findings, adding to the current discussion on the role of pHF. One of them is the GINI study, a large, well-designed and conducted, randomized, double-blind (until 3 years of age) trial, with a 15-year follow-up period [22–24]. Second, also a large, but single-blinded study is the MACS study [17]. A number of issues related to these two trials have been discussed by the authors themselves elsewhere [28–30].

We present the results of both the ITT (more precisely, available case analysis) and the per-protocol analyses, as they complement each other. In the two largest studies (i.e., the GINI and the MACS), the rate of breastfeeding was high (approximately 40%). Thus, the ITT analyses included infants who were exclusively breastfed, including infants who were never exposed to pHF. The per-protocol analysis included all participants who adhered adequately to the assigned regimen. While not ideal from a methodological point of view, the per-protocol analysis is important for understanding the role of pHF in allergy prevention, and hence, our decision to include both. Clearly, the promotion of exclusive feeding with pHF from birth would be unethical.

We focused only on a single formula. This decision was in line with the opinion of the European Food Safety Authority [31], which has clearly stated that it is necessary to demonstrate if, and to what extent, *a particular* formula reduces the risk of developing short- and long-term clinical manifestations of allergy in at-risk infants who are not breastfed.

### Strengths and limitations

The review question and inclusion criteria were clearly defined. Various major databases were accessed. No language restrictions were applied. The corresponding authors were contacted to clarify reported data in the case of questions. Experts in the field were contacted. Unpublished data from the manufacturer of pHF were available. Thus, the risk that relevant studies were missed was minimized. Additionally, efforts were made to minimize reviewers’ errors and bias. Two reviewers independently identified, selected, and assessed the risk of bias using accepted criteria in the included trials. Another strength is the use of the GRADE profile to rate the overall quality of evidence, which can be useful for future guideline development.

However, this review has some limitations. As this review represents an update of our previously reported meta-analysis [7], the analyses were defined a priori*.* However, the protocol of the review has not been registered. This was because the review was carried out according to the same methodology as used in our original review. Not all included trials were free of the risk of bias. Only the GINI study seemed methodologically sound. One concern with studies involving hydrolyzed formulas is the lack of true blinding. The latter is challenging, as hydrolyzed formulas have a specific taste and smell, and study personnel and caregivers may have suspected the intervention. Assessment of selective reporting was challenging, as with one exception, the trial protocols were not registered. Only the MACS was registered, but retrospectively. However, registration on a public trial registration database prior to the start of the study with sufficient protocol information has become the standard only recently.

The majority of included studies were industry supported. A 2012 Cochrane Review provided evidence that there is bias associated with study funding sources [32]. Compared with non–industry-sponsored studies, drug and medical device studies sponsored by the manufacturers tended to have more favorable effectiveness and harm findings and more favorable conclusions. Funding of research by manufacturers of infant formulas may be considered an even more complex and controversial topic because of the need for protection and promotion of breastfeeding. However, in the case of studies involving infant formulas, industry involvement is unavoidable, as investigators lack the means to manufacture quality infant products. Thus, industry sponsorship will likely continue to be a major source of funding for research on infant formulas, although collaborative clinical research between academia and industry is in both the mutual and public interest. Of note, in the largest GINI study, even if the study formulas were provided by the manufacturers (for the first 3 years), the study was financed from public resources.

Ideally, the diagnosis of allergic diseases should be based on widely agreed-upon criteria. However, in most of the included studies, heterogeneous definitions made direct comparisons between the studies difficult. Caution is needed when interpreting ‘all allergic diseases’ [33]. In the included trials, this composite outcome was defined differently by the authors of the original trials. It is also important to consider how the outcomes were assessed. For example, in one of the studies, the primary outcome measures, including eczema and any allergic manifestation, were assessed during telephone interviews with parents [17]. This contrasts with the assessment in another trial, at least during the first 3 years of the study, made by one investigator according to predefined diagnostic criteria and confirmation by a second, specially trained allergist [22].

With two exceptions [17, 22], the included trials had small sample sizes and lacked sample size calculations. However, to increase power is one of the reasons why a meta-analysis is performed within a systematic review [34].

While not formally assessed due to the limited number of eligible trials for any given outcome, publication bias, i.e., bias due to the publication or non-publication of research findings depending on the nature and direction of the results, cannot be excluded.

Finally, one of the limitations of our review is that we did not systematically assess the safety of pHF. All formulas intended for infants must be safe and suitable to meet their nutritional requirements; they must promote the growth and development of infants born at term when used as the sole source of nutrition during the first months of life, as well as when used as the principal liquid element in a progressively diversified diet after the introduction of appropriate complementary feeding [31]. However, available data do not indicate that pHFs are potentially harmful for healthy, term infants. Based on limited available data, summarized elsewhere, the use of pHF in healthy infants is safe with regard to growth [35].

### Agreement and disagreement with other studies or reviews

Compared with a 2016 meta-analysis by Boyle et al. [6], our review focuses on one type of hydrolyzed formula (pHF), as not all hydrolyzed formulas are equal. Moreover, our review included only RCTs, and it excluded observational studies. Compared with the analysis by Boyle et al., we included only studies carried out in a high-risk population. We report outcomes at time intervals reported by the authors of the original studies. In contrast, Boyle et al. grouped participants who were aged at assessment 0–4, 5–14, and ≥15 years. As the reviewers subjectively chose these time intervals, a number of decisions were made as to which data should be used for their analyses, which may have introduced bias. Both reviews presented the intention-to-treat analyses without imputation. However, Boyle et al. presented the results of systematic reviews as odds ratios (ORs), mainly because the GINI study used generalized estimating equations (GEE) (to generate odds ratios in some of their publications). Consequently, ORs were calculated by the authors for all data pooled with GINI GEE data. In our review, we present the RR, which is recommended by the Cochrane Handbook for Systematic Reviews of Interventions as the summary statistic that is easier to understand and apply in practice. We contacted the study authors to obtain additional information if it was not available from the published report or reports from studies. Furthermore, Boyle et al. included studies in which in the intervention group, but not in the control group, additional interventions were applied such as house dust mite control measures and a smoke-free environment. In our analysis, we excluded such studies. Moreover, Boyle et al. pooled data on cumulative incidence and prevalence. In our analysis, we report these data separately. Of note, in the Food Standards Agency report, post hoc*,* Boyle et al. evaluated the effect of using pHF, as in our review, compared with cow’s milk-based formula on the risk of eczema in children aged 0–4 years (https://www.food.gov.uk/science/research/allergy-research/fs305005hf). No difference between the groups was found. However, it remains unclear which studies were included in the pooled analysis. Taken together, in our view, our results more precisely define the effects of pHF on allergy outcomes.

## Conclusions

Our systematic review was designed to resolve uncertainty with regard to the use of pHF and the risk of allergic disease. Both ITT analyses and per-protocol analyses showed that the reduction was statistically significant at some, albeit not all, time points. We confirmed our earlier conclusion that there is evidence to consider use of pHF as an alternative to CMF as an option for reducing the risk of allergy, particularly eczema; however, the certainty of the evidence is low. One characteristic that makes our meta-analysis distinct from other reviews is that it focuses exclusively on only one type of pHF. Hydrolyzed formula manufactured using different methods needs to be evaluated separately and such analyses are underway. Further studies of use of pHF for allergy reduction are needed to clarify populations of infants most likely to benefit. In the meantime, combining raw data from individual trials via an individual participant data meta-analysis can yield more reliable estimates of treatment effects with universal applicability.

## Additional files

Additional file 1: Review protocol. (DOC 77 kb)

Additional file 2: Data S1. Supplementary information on review methods, including electronic searches, data collection and analysis, and data extraction and management. **Figure S1.** Identification process for eligible studies. **Figgure S2.** Risk of bias graph: review authors’ judgements about each risk of bias item presented as percentages across all included studies. **Figure S3.** Risk of bias summary: review authors’ judgements about each risk of bias item for each included study. **Table S1.** Characteristics of the included studies. **Table S2.** Characteristics of the excluded studies involving partially hydrolyzed 100% whey formula. **Table S3.** GRADE evidence profile summarizing the effect of partially hydrolyzed formula (pHF) vs. cow’s milk formula (CMF) on eczema. **Table S4.** GRADE evidence profile summarizing the effect of partially hydrolyzed formula (pHF) vs. cow’s milk formula (CMF) on all allergic diseases. (DOC 211 kb)

## Abbreviations

CMF: Cow’s milk formula
NNT: Number needed to treat
pHF: Partially hydrolyzed 100% whey formula
RCT: Randomized controlled trial
