# Supplementary material for: A partially hydrolyzed 100% whey formula and the risk of eczema and any allergy: an updated meta-analysis
Source: World Allergy Organ J. 2017 Jul 26;10(1):27. doi: 10.1186/s40413-017-0158-z (PMC5527395; doi:10.1186/s40413-017-0158-z)
Supplement: Supplementary file 1 — Review protocol. (DOC 77 kb) [file 40413_2017_158_MOESM1_ESM.doc]

**A partially hydrolyzed 100% whey formula and the risk of eczema and any allergy: protocol of an updated meta-analysis**

[This systematic review will be an update of previously reported meta-analysis:

*Szajewska H, Horvath A. Curr Med Res Opin 2010;26:423-437].*

| **Title** | A partially hydrolyzed 100% whey formula and the risk of eczema and any allergy: an updated meta-analysis |
| --- | --- |
| **Review team details** | H Szajewska, A Horvath  The Medical University of Warsaw,  Zwirki i Wigury 63A, 02-091 Warsaw, Poland  email: hania@ipgate.pl |
| **Review question** | In healthy infants at high risk for allergy, do partially hydrolyzed 100% whey formula compared with a standard infant formula reduce the risk of allergy? |
| **Searches** | The following search terms will be used: ("infant, newborn"[MeSH Terms] OR ("infant"[All Fields] AND "newborn"[All Fields]) OR "newborn infant"[All Fields] OR "neonat*"[All Fields] OR "infant"[MeSH Terms] OR "infant"[All Fields] OR pediatric[All Fields] OR paediatric[All Fields]) AND ("hypersensitivity"[MeSH Terms] OR "hypersensitivity"[All Fields] OR "allergy"[MeSH Terms] OR "allergy"[All Fields] OR "allergy and immunology"[All Fields] OR "food allergy"[All Fields] OR "milk allergy"[All Fields] OR "eczema"[MeSH Terms] OR "eczema"[All Fields] OR "wheezing"[All Fields]) OR "asthma"[MeSH Terms] OR "asthma"[All Fields]) AND ([feed* OR food OR formula[All Fields] OR hydrolysed [All Fields] OR hydrolyzed [All Fields] OR "diet"[MeSH Terms] OR "diet"[All Fields] OR milk*]). For searching, the names under which pHF is marketed in various countries (Beba-HA®, Good Start®, NAN-HA®, Nidina®) will be utilized.  Additionally, reference lists from identified studies, key review articles, previous systematic reviews that assessed the effects of hydrolyzed formulas in infants, and authors’ files will be searched. Experts in the field will be contacted for additional references. Certain publication types (i.e., letters to the editor, abstracts, proceedings from scientific meetings) will be excluded, unless a full set of data may be obtained from the authors.  The search will be carried out independently by 2 reviewers. |
| **Participants/ population** | Healthy term infants at high risk of developing allergy, as assessed by a family history (the presence of allergy in at least one parent and/or sibling) and/or other markers (as determined by the study investigators). |
| **Intervention(s), exposure(s)** | A partially hydrolyzed 100% whey formula. |
| **Comparator(s)/ control** | Subjects in the control group will receive standard cow’s milk infant formula. If other experimental arms will be available, they will not considered |
| **Types of study to be included** | Randomized controlled trials & quasi-randomized controlled trials (i.e. trials in which allocation may be based on the person’s date of birth, the person’s medical record number, or the day of the week or month of the year). |
| **Context** | Recently, the role of using hydrolyzed formula for the prevention of allergic disease has been questioned. However, not all hydrolyzed formulas are equal. The efficacy of each hydrolyzed formula should be established separately. |
| **Primary outcome(s)** | 1. Eczema (the results of previous systematic reviews showed that, if there is an effect of hydrolyzed formulas, it is the reduction of the risk of eczema) 2. All allergic disease (as defined by the authors of original publications)   Both outcomes at time intervals reported by the authors of the original publications |
| **Data extraction, (selection and coding)** | Two reviewers will perform data extraction, using standard data-extraction forms. Data on the methods, participants, interventions, outcomes, sample size calculation, and the funding of each study will be extracted. For dichotomous outcomes, the total number of participants and the number of participants who experienced the event will be extracted. For continuous outcomes, the total number of participants and the means and standard deviations will be extracted if provided by the authors. If not, we will present data as reported by the authors of the original papers. If feasible, the data will be entered into the Review Manager (RevMan) for analysis. |
| **Risk of bias (quality) assessment** | The reviewers independently, but without being blinded to the authors or journal, will assess the risk of bias in the studies that meet the inclusion criteria. The Cochrane Collaboration tool for assessing risk of bias will be used, which includes the following: type of randomization method (selection bias), allocation concealment (selection bias), blinding of participants and personnel (performance bias), blinding of outcome assessment (detection bias), incomplete outcome data (attrition bias), selective reporting (reporting bias), and other bias (defined as co-interventions and compliance).  For the overall quality (certainty) of evidence for each estimate for each individual outcome, we will use the Grading of Recommendations, Assessment, Development and Evaluation (GRADE) approach, which includes assessing within study risk of bias (methodological quality), directness of evidence, heterogeneity, precision of effect estimates, and risk of publication bias. |
| **Assessment of heterogeneity** | Heterogeneity will be quantified by 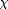^2^ and *I*^2^, which can be interpreted as the percentage of the total variation between studies that is attributable to heterogeneity rather than to chance. A value of 0% indicates no observed heterogeneity, and larger values show increasing heterogeneity. If heterogeneity is not revealed, the fixed effects models will be presented. If there is substantial heterogeneity (over 50%), the random effects models will be presented. |
| Assessment of reporting biases | A test for asymmetry of the funnel plot will be performed, if sufficient (≥10) eligible trials are available for any given outcome. |
| **Strategy for data synthesis** | We will attempt a meta-analysis using standard Cochrane methodology as described in the Cochrane Handbook for Systematic Reviews of Interventions (Higgins 2011). The data will be analyzed using Review Manager (RevMan)  The data will be pooled using:   1. intention-to-treat analysis, i.e., an analysis in which data are analyzed for every participant for whom the outcome was obtained (also known as available case analysis); 2. per-protocol analysis, i.e., analysis which includes all participants who adhered adequately to the assigned regimen. |
| **Dissemination plans** | Peer reviewed journal article, and an abstract at local, national or international conferences. |
| **Anticipated start date** | April 2016 |
| **Anticipated completion date** | August 2016 |
| **Funding sources/sponsors** | This systematic review will be carried out as part of research activities of the authors, and thus funded in full by The Medical University of Warsaw. |
| **Conflict of interest** | HS has participated as a clinical investigator, and/or advisory board member, and/or consultant, and/or speaker for companies manufacturing infant formulas, i.e., Arla, Danone, HiPP, Nestle, Nestle Nutrition Institute, Nutricia, and Mead Johnson.  AH has participated as a speaker for companies manufacturing infant formulas, i.e., Danone, Nestle, Nestle Nutrition Institute, Nutricia, and Mead Johnson. |
