# Supplementary material for: A partially hydrolyzed 100% whey formula and the risk of eczema and any allergy: an updated meta-analysis
Source: World Allergy Organ J. 2017 Jul 26;10(1):27. doi: 10.1186/s40413-017-0158-z (PMC5527395; doi:10.1186/s40413-017-0158-z)
Supplement: Supplementary file 2 — Supplementary information on review methods, including electronic searches, data collection and analysis, and data extraction and management. Figure S1. Identification process for eligible studies. Figgure S2. Risk of bias graph: review authors’ judgements about each risk of bias item presented as percentages across all included studies. Figure S3. Risk of bias summary: review authors’ judgements about each risk of bias item for each included study. Table S1. Characteristics of the included studies. Table S2. Characteristics of the excluded studies involving partially hydrolyzed 100% whey formula. Table S3. GRADE evidence profile summarizing the effect of partially hydrolyzed formula (pHF) vs. cow’s milk formula (CMF) on eczema. Table S4. GRADE evidence profile summarizing the effect of partially hydrolyzed formula (pHF) vs. cow’s milk formula (CMF) on all allergic diseases. (DOC 211 kb) [file 40413_2017_158_MOESM2_ESM.doc]

**Additional file 2**

**A partially hydrolyzed 100% whey formula and the risk of eczema and any allergy: an updated meta-analysis**

**Short title: Partially hydrolyzed whey formula in allergy prevention**

#### DATA, TABLES & FIGURES LEGENDS

**Data S1.** Supplementary information on review methods, including electronic searches, data collection and analysis, and data extraction and management.

**Figure S1.** Identification process for eligible studies.

**Figure S2.** Risk of bias graph: review authors' judgements about each risk of bias item presented as percentages across all included studies.

**Figure S3.** Risk of bias summary: review authors' judgements about each risk of bias item for each included study.

**Table S1.** Characteristics of the included studies.

**Table S2.** Characteristics of the excluded studies involving partially hydrolyzed 100% whey formula.

**Table S3.** GRADE evidence profile summarizing the effect of partially hydrolyzed formula (pHF) vs. cow’s milk formula (CMF) on eczema.

**Data S1.** Supplementary information on review methods, including electronic searches, data collection and analysis, and data extraction and management.

#### SEARCH METHODS FOR IDENTIFICATION OF STUDIES

**Electronic searches**

The Cochrane Central Register of Controlled Trials (CENTRAL, the Cochrane Library), MEDLINE, and EMBASE databases were searched for relevant studies from September 2009 (end date of last search) to June 2016. There were no language restrictions. As in our previous review, the search strategy included use of a validated filter for identifying controlled trials, which was combined with a topic-specific strategy.

In brief, the following search terms were used: ("infant, newborn"[MeSH Terms] OR ("infant"[All Fields] AND "newborn"[All Fields]) OR "newborn infant"[All Fields] OR "neonat*"[All Fields] OR "infant"[MeSH Terms] OR "infant"[All Fields] OR pediatric[All Fields] OR paediatric[All Fields]) AND ("hypersensitivity"[MeSH Terms] OR "hypersensitivity"[All Fields] OR "allergy"[MeSH Terms] OR "allergy"[All Fields] OR "allergy and immunology"[All Fields] OR "food allergy"[All Fields] OR "milk allergy"[All Fields] OR "eczema"[MeSH Terms] OR "eczema"[All Fields] OR "wheezing"[All Fields]) OR "asthma"[MeSH Terms] OR "asthma"[All Fields]) AND ([feed* OR food OR formula[All Fields] OR hydrolysed [All Fields] OR hydrolyzed [All Fields] OR "diet"[MeSH Terms] OR "diet"[All Fields] OR milk*]). For searching, the names under which pHF is marketed in various countries (Beba-HA®, Good Start®, NAN-HA®, Nidina®) were also utilized. The search was carried out independently by 2 reviewers.

**Searching other resources**

We searched reference lists from identified studies, key review articles, previous systematic reviews that assessed the effects of hydrolyzed formulas in infants, and authors’ files. The Nestle´ Nutrition Institute (NNI) provided us with all unpublished data from clinical trials with the pHF. Experts in the field were contacted for additional references. Clarification of outcome data was requested (and obtained) by email from the corresponding authors of 2 trials and one meta-analysis. Certain publication types (i.e., letters to the editor, abstracts, proceedings from scientific meetings) were excluded, unless a full set of data was obtained from the authors.

## DATA COLLECTION AND ANALYSIS

### Selection of studies

Two reviewers initially screened the title, abstract, and keywords of every record identified with the search strategy, and they retrieved the full text of potentially relevant trials and of records for which the relevance was unclear. Same reviewers independently applied the inclusion criteria to each potentially relevant trial to determine its eligibility. If differences in opinion existed, they were resolved by discussion until a consensus was reached.

**Data extraction and management**

Data extraction was performed using standard data-extraction forms. In addition to data such as methods, participants, interventions, and outcomes, we collected information about sample size calculation and the funding of each study. One reviewer (A.H.) extracted the data from the included studies, and the second author (H.S.) checked the extracted data. Discrepancies between the reviewers were resolved by discussion until a consensus was reached. Foreign language studies were translated. Participants, interventions, comparisons, and outcomes were taken into consideration to determine whether they were similar enough to allow pooling.

### Assessment of risk of bias in included studies

### Type of randomization method (selection bias), allocation concealment (selection bias), blinding of participants and personnel (performance bias), blinding of outcome assessment (detection bias), incomplete outcome data (attrition bias), selective reporting (reporting bias), and other bias (defined as co-interventions and compliance) were considered.

#### Figure S1. Identification process for eligible studies.

3 database searches

2910 Medline

2143 Embase

445 Central

54 abstracts reviewed

- only clinical trials
- no reviews, systematic reviews or meta-analysis

8 abstracts excluded

- no full publication avaiable^17,21,33,37,45,50,56,57^

46 full articles reviewed

33 articles excluded

- 14 Non-RCT^18,19,28-31,36,38-42,47,54^
- 6 RCT – no outcomes at selected time points^32,35,43,46,48,53^
- 2 RCT – complex intervention introduced (non pure pHF)^20,26^
- 4 RCT – intervention group use a different type pHF^27,44,49^ or eHF^52^
- 4 RCT – study data have been questioned ^22-25^
- 1 RCT – study conducted in low-risk population^34^
- 1 Duplicate publication^55^
- 1 Report provided by NNI provided also as full paper^51^

13 full articles included^4-16^

- 8 independent RCTs^4-9.11,16^
- 1 study – described follow-up the same populations^9-10^
- 4 studies – described follow-up the same populations^11-15^

**Figure S2.** Risk of bias graph: review authors' judgements about each risk of bias item presented as percentages across all included studies.

**
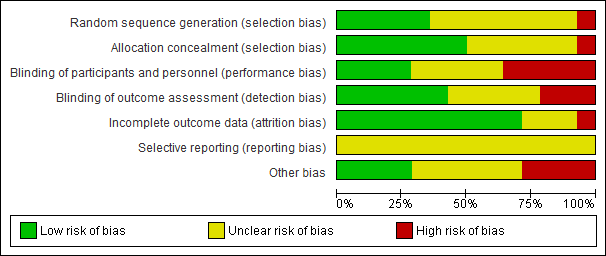
**

**Figure S3.** Risk of bias summary: review authors' judgements about each risk of bias item for each included study.

**
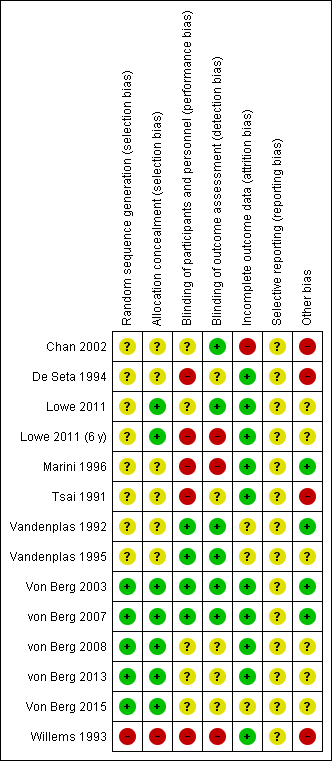
**

**Table S1. Characteristics of the included studies.**

| Study | Population (risk of allergy) | Intervention (pHF)  vs. control (CMF)  N/n | Duration of  intervention | Recommendation for complementary feeding | Co-intervention* | Follow up | Definition of allergic manifestations | Sample size | Funding |
| --- | --- | --- | --- | --- | --- | --- | --- | --- | --- |
| Chan 2002 (Singapore)[[1]](#endnote-2) | Asthma, eczema, or allergic rhinitis in a first-degree relative | 76/77 | First 4 mo, exclusively study formula;  >4 mo formula continuation up to 12 mo | After 4 mo weaning diet with no restrictions | No | 30 mo | **Atopic dermatitis**  Eczematous eruption that was pruritic, had a typical morphology and distribution, with a tendency towards chronicity or recurrence.  **Wheezing**  An audible high-pitched expiratory sound in association with coughing and breathlessness; reversible with bronchodilators  **Urticaria**  Rash with a typical morphology, reported by a parents and/or confirmed by a physician | Not reported | Research grant from Nestle Singapore (Pte) Ltd. |
| De Seta1994 (Italy)[[2]](#endnote-3) | One 1st degree relative with asthma, rhinitis, conjunctivitis, eczema, or cow’s milk protein intolerance | 23/39 | 6 mo (exclusively) | Cow’s milk protein, eggs, poultry, fish >6 months | No | 24 mo | **AD/Atopic eczema**  Eczema diagnosis made according to the Hanifin and Rajka criteria.  **Cow’s milk protein intolerance with GI manifestation**  Vomiting and/or diarrhea occurred after ingestion of an offending food, on at least two occasions with concomitant food specific IgE.  **Asthma**  **Probable** – when 2 & **Definite** – when 3 episodes of physician documented wheezing occurred unassociated with foreign body, congenital abnormality, croup, aspiration or GERD.  **Allergic disease** – definition not provided | Not reported | Not reported |
| Lowe 2011 (Australia)[[3]](#endnote-4) | Representative population; 1st degree relative with a history of eczema, asthma, allergic rhinitis, food allergy | 206/206 | 12 mo | Introduction of rice cereal, pureed apple, and pear was recommended from  4 months of age, and vegetables and other fruit from 6 months. Meats were  introduced from 8 months, and nonrice cereals from 9 months. Dairy products,  egg, fish, peanut, and nuts were avoided until 12 mo of age | No | 24 mo | **AD/Eczema**  Doctor-diagnosed eczema or any rash that was treated with topic steroid preparation (excluding rash that only affected the scalp or nappy region), assessed during telephone interviews with parents.  **Food reaction**  An acute skin rash (urticarial, angioedema, erythematous), a flare of pre-existing eczema, signs of anaphylaxis, or vomiting observed within 2 hours after ingestion of food.  **Any allergic manifestation**  Presence of eczema or food reaction within the first years of life. | Correctly calculated | Nestec Ltd., a subsidiary of Nestle Australia, provided the study formulas and staff funding for the first 6 years of the study. |
| Marini 1996 (Italy)[[4]](#endnote-5) | Well-defined family history of allergy (urticarial, angioedema, atopic dermatitis, hay fever, asthma, anaphylaxis) in either parent | 48/47 | BF and the study formula up to 5 mo | Cow’s milk and allergenic foods avoided to 1 yr;  All foods introduced at 12 mo | Avoidance of parental smoking in the presence of babies; day care >2 y of life | 36 mo | **Atopic dermatitis**  Areas of scaly, erythematous, and itchy eczematous rash primarily involving the face and scalp, behind the ears, and flexural folds. Only eczema localized to at least two typical areas was recorded.  **Recurrent wheezing**  A history of at least three episodes of expiratory wheezing and confirmed by a physician.  **Urticaria**  Symptoms appeared at least twice within 1 h after exposure to a particular antigen.  **GI symptoms**  Vomiting and/or diarrhea after excluding ordinary feeding problems, coincidental infection and lactose intolerance; after elimination of suspected food – diagnosis was confirmed by open challenge.  **Allergic rhinitis**  At least three consecutive weeks of rhinorrhoea eventually associated with conjuctival symptoms without infectious features and without simultaneous rhinorrhoea in household contacts. | Not reported | Not reported |
| Tsai 1991 (Taiwan)[[5]](#endnote-6) | Family allergy based on Family History of Allergy Score score >3 | 15/18 | Intervention group: BF 1-2 mo; pHF up to 6 mo  Control group: CMF during first 6 mo | Not reported | No co-interventions reported. | 12 mo | **Atopic dermatitis**  AD mild: lesions involved forehead or cheek that needed no treatment; moderate lesions involving face and extensor surface that needed treatment except steroids; severe lesions involving face, extensor surface, thigh and abdomen that needed local or even systematic steroid treatment.  **Allergic symptoms** (allergic disease, asthma, urticaria, GI symptoms, eczema, protein intolerance) were not specifically defined. Diagnosis was based on history and physical examination performed by a physician. Total serum IgE, specific IgE and SPT were evaluated. | Not reported | Formula provided by ANPING Ltd. |
| Vandenplas 1992, 1995 (Belgium)[[6]](#endnote-7) [[7]](#endnote-8) | At least 2 first- degree relatives with atopy | 32/35 | 6 mo | Apple from 4 mo; “normal” diet after 6 months | No | Up to 5 y | **Atopic dermatitis**  Eczematous eruption with at least three of the four following criteria: (1) pruritis; (2) typical morphology and distribution; (3) chronicity or recurrence of the symptoms; (4) specific IgE at the time the rash is present.  **Wheezing**  Cough and other respiratory symptoms lasted longer than 24 hours in the absence of fever and unassociated with infection.  **Chronic rhinitis**  A clear water discharge form the nose  **GI symptoms**  Diagnosis of vomiting and/or diarrhea was based on a history and typical symptoms, after excluding infection.  Diagnosis was supported by laboratory tests (IgE, RAST, IgG4) and SPT (in infants with symptoms < 6 mo).  **Allergic manifestations** – definition not provided | Not reported | Nestle provided formula and performed statistical analysis. |
| Von Berg 2003, 2007, 2008, 2013, 2016 (Germany)[[8]](#endnote-9) [[9]](#endnote-10) [[10]](#endnote-11) [[11]](#endnote-12) [[12]](#endnote-13) | At least one first-degree family member with allergy | 557/556 | In addition to BF, study formula provided until infant 6 mo of age | Solid food after study period – add one food a week, avoid common allergenic foods (e.g. milk, dairy products, hen’s eggs, soy, fish, nuts, tomatoes, citrus fruit) in 1st year of life. | BF for at least 4 mo, preferably 6 mo. No dietary restrictions during BF. | Up to 15 years | **Atopic dermatitis**  Typical morphology and distribution of skin lesions (face, neck, and scalp, flexural folds, hands, and extensor sides of the extremities); pruritis, and tendency toward chronicity. The morphologic diagnosis was confirmed by a trained second physician.  **Urticaria**  At least 2 episodes of itching eruptions or swelling with typical appearance, caused by the same allergen; supported by SPT (a wheal ≥3 mm) or specific IgE ≥0.35 KU/L) or positive oral challenge  **Food allergy with GI manifestation**  Symptoms such as: bloodstained stools, diarrhea, vomiting, frequent regurgitation, colicky behavior, failure to thrive not explained by any other condition;  Diagnosis was supported by oral food challenge.  **Asthma (children > 3ys)**  ≥ 3 episodes of wheezing or nighttime cough without infection, or regular use of asthma medication for obstructive symptoms.  **Rhinitis**  A problem with sneezing or a runny or blocked nose without cold or flu.  A specific IgE – screening test for the most common food and inhalalnt allergenes was measured.  **Allergic manifestations** diagnosed at 12 months as AD, urticaria, FA | Correctly calculated | For 3 y, the Federal ministry for Education, Science, Research and Technology and the Child Health Research Foundation.  The 3-6 and 10-year follow up, study centers funding. The 15-year follow up, Mead Johnson and Nestle, and the European Studies (e.g. MeDALL, ESCAPE).  Formula provided by Nestle, Hipp, Milupa, and Mead Johnson. |
| Willems 1993 (Belgium) [[13]](#endnote-14) | Family history of allergy in at least 1 family member and cord IgE ≥0.5 IU/L | 55/67 | 3 mo (exclusively) | >3 mo unrestricted diet | No | 12 mo | **AD/Eczema**  Typical erythematopapulovesicular lesions in typical areas excluding seborrhea  **Allergic symptoms** (eczema, asthma, recurrent episodes of bronchitis, persistent rhinitis, persistent GI symptoms, serious sleeping difficulties) were not specifically defined. | Not reported | Unclear  Co-investigator from FNRS Brussels |

AD: atopic dermatitis; BF: breastfeeding; CM: cow's milk; CMF: cow’s milk formula; ITT: intention-to-treat analysis; pHF: partially hydrolyzed formula;

* Co-interventions included environmental modifications (e.g., avoidance of house dust mite, modifications of the diet of mothers)

**Table S2. Characteristics of the excluded studies involving partially hydrolyzed 100% whey formula.**

|  | **Study** | **Reasons for exclusion** |
| --- | --- | --- |
| 1 | Akimoto 1997[[14]](#endnote-15) | Abstract. Not an RCT; cohort study. |
| 2 | Barberi 1993[[15]](#endnote-16) | Not an RCT. |
| 3 | Bardare 1993[[16]](#endnote-17) | Not an RCT; intervention – BF vs. pHF. |
| 4 | Becker 2004[[17]](#endnote-18) | RCT. In the intervention group, use of a multifaceted intervention program (house dust mite control measures, pet avoidance measures, smoke-free environment for the infant, breastfeeding, and pH whey formula). In the control group, the usual care provided by the primary physicians. |
| 5 | Boyle 2014[[18]](#endnote-19) | Abstract; RCT; intervention – pHF-whey combined with specific mixture of prebiotic oligosaccharides vs. SF |
| 6-9 | Chandra 1989[[19]](#endnote-20) 1991[[20]](#endnote-21) 1992[[21]](#endnote-22) 1997[[22]](#endnote-23) | RCT. Co-authored by the investigator whose data have been questioned. |
| 10 | Chan-Yeung 2000[[23]](#endnote-24) | RCT. In the intervention group, use of a multifaceted intervention program (house dust mite control measures, pet avoidance measures, smoke-free environment for the infant, breastfeeding, and pH whey formula). In the control group, the usual care provided by the primary physicians. |
| 11 | Chirico 1997[[24]](#endnote-25) | RCT; intervention pHF (Vivena HA) vs. SF. |
| 12 | D’Agata 1996[[25]](#endnote-26) | Not an RCT; intervention – pHF vs. SF vs. soy product. |
| 13-15 | Exl 1998,[[26]](#endnote-27) 2000[[27]](#endnote-28), 2000[[28]](#endnote-29) | Not an RCT; conducted in non-risk population. |
| 16 | Halken 2000[[29]](#endnote-30) | qRCT; intervention – pHF-whey vs. SF; no outcomes at selected time points. |
| 17 | Hartman 1994[[30]](#endnote-31) | Abstract. RCT; conducted in non-risk population. |
| 18 | Iikura 1995*[[31]](#endnote-32)* | RCT; conducted in non-risk population (later revealed that 60% had a family history of allergy). |
| 19 | Lam 1992[[32]](#endnote-33) | RCT; compared CMF vs. pHF-w; no outcomes at selected time points. |
| 20 | Laforgia 1996[[33]](#endnote-34) | Not an RCT; cohort study; intervention – pHF vs. BF. |
| 21 | Mautone 1991[[34]](#endnote-35) | Abstract. Not an RCT; cohort study; intervention – pHF vs. BF. |
| 22 | Macagno 1989[[35]](#endnote-36) | Not an RCT; prospective cohort study; intervention – pHF vs. BF. |
| 23 | Marini 1990[[36]](#endnote-37) | Not an RCT; prospective cohort study; intervention – pHF vs. BF. |
| 24 | Marini 1990[[37]](#endnote-38) | Not an RCT; prospective cohort study; follow-up the same population as Marini^23^ |
| 25 | Marini 1990 (unpublished)[[38]](#endnote-39) | Reported as ‘partly sequential’. |
| 26 | Martinez-Valvere 1993[[39]](#endnote-40) | Not an RCT; no definitions of allergic symptoms reported. |
| 27 | Nentwich 2001[[40]](#endnote-41) | qRCT; intervention – pHF-whey vs. CMF; no outcomes at selected time points. |
| 28 | Oldaeus 1997[[41]](#endnote-42) | RCT; intervention – eHF vs. pHF (HA, Mead Johnson) vs. SF. |
| 29 | Porch 1996[[42]](#endnote-43) | Abstract. The same population as included in study by Porch 1998. |
| 30 | Porch 1998[[43]](#endnote-44) | RCT; intervention – pHF-whey vs. CMF; no outcomes at selected time points. |
| 31 | Schmidt 1995[[44]](#endnote-45) | Not an RCT; observational study (infants allocated to formula at parents' discretion). |
| 32 | Schmitz 1992[[45]](#endnote-46) | RCT; intervention – CMF vs. pHF-whey only for the first 5 days |
| 33 | Shao 2006[[46]](#endnote-47) | RCT; intervention – CMF vs. pHF-whey (no HA, Nestle) |
| 34 | Silva Rey 1996[[47]](#endnote-48) (thesis) | Not an RCT. |
| 35 | Tsai 1991[[48]](#endnote-49) | Report provided by Nestle Nutrition Institute. Available also as full paper. |
| 36 | Vaarla 2012[[49]](#endnote-50) | RCT; intervention – CMF vs. eHF-whey. |
| 37 | Vandenplas 1988[[50]](#endnote-51) | RCT; intervention – CMF vs. pHF-whey; no outcomes at selected time points. |
| 38 | Vandenplas 1989[[51]](#endnote-52) | Retrospective study. |
| 39 | Vandenplas 1992[[52]](#endnote-53) | Duplicate publication. |
| 40 | Vassella 1994[[53]](#endnote-54) | Abstract. Not an RCT; observational study (infants allocated to feeding group at parents' discretion); intervention – pHF vs. BF. |
| 41 | Wen[[54]](#endnote-55) | Not an RCT; prospective study. |

BF: breastfed; CMF: cow’s milk formula; eHF: extensively hydrolyzed formula; HA: hypoallergenic; pHF: partially hydrolyzed formula; RCT: randomized controlled trial.

**Table S3.** GRADE evidence profile summarizing the effects of partially hydrolyzed formula (pHF) vs. cow’s milk formula (CMF) on eczema.

| **Quality assessment** | | | | | | | **№ of patients** | | **Effect** | | **Quality** |
| --- | --- | --- | --- | --- | --- | --- | --- | --- | --- | --- | --- |
| **№ of studies** | **Study design** | **Risk of bias** | **Inconsistency** | **Indirectness** | **Imprecision** | **Other considerations** | **aa** | **bb** | **Relative (95% CI)** | **Absolute (95% CI)** |
| Eczema CUMULATIVE INCIDENCE ITT - Eczema up to 1 y ITT | | | | | | | | | | | |
| 4 | randomised trials | not serious | serious ^a^ | not serious | serious ^b^ | none | 129/717 (18.0%) | 154/741 (20.8%) | **RR 0.75** (0.49 to 1.15) | **52 fewer per 1 000** (from 31 more to 106 fewer) | ⨁⨁◯◯ LOW |
| Eczema CUMULATIVE INCIDENCE ITT - Eczema up to 2 y ITT | | | | | | | | | | | |
| 2 | randomised trials | not serious | serious ^a^ | not serious | serious ^b^ | none | 105/244 (43.0%) | 108/250 (43.2%) | **RR 0.80** (0.37 to 1.73) | **86 fewer per 1 000** (from 272 fewer to 315 more) | ⨁⨁◯◯ LOW |
| Eczema CUMULATIVE INCIDENCE ITT - Eczema up to 3 y ITT | | | | | | | | | | | |
| 3 | randomised trials | not serious | not serious | not serious | serious ^c^ | none | 121/461 (26.2%) | 171/539 (31.7%) | **RR 0.82** (0.68 to 1.00) | **57 fewer per 1 000** (from 0 fewer to 102 fewer) | ⨁⨁⨁◯ MODERATE |
| Eczema CUMULATIVE INCIDENCE ITT - Eczema up to 5-6 y ITT | | | | | | | | | | | |
| 2 | randomised trials | not serious | not serious | not serious | serious ^c^ | none | 142/462 (30.7%) | 177/476 (37.2%) | **RR 0.83** (0.69 to 0.99) | **63 fewer per 1 000** (from 4 fewer to 115 fewer) | ⨁⨁⨁◯ MODERATE |
| Eczema CUMULATIVE INCIDENCE ITT - Eczema up to 10 y ITT | | | | | | | | | | | |
| 1 | randomised trials | not serious | not serious | not serious | serious ^b^ | none | 197/557 (35.4%) | 225/556 (40.5%) | **RR 0.87** (0.75 to 1.02) | **53 fewer per 1 000** (from 8 more to 101 fewer) | ⨁⨁⨁◯ MODERATE |
| Eczema CUMULATIVE INCIDENCE ITT - Eczema up to 15 y ITT | | | | | | | | | | | |
| 1 | randomised trials | not serious | not serious | not serious | very serious ^b,d^ | none | 206/557 (37.0%) | 235/556 (42.3%) | **RR 0.88** (0.76 to 1.01) | **51 fewer per 1 000** (from 4 more to 101 fewer) | ⨁⨁◯◯ LOW |
| Eczema PERIOD PREVALENCE ITT - Eczema at 1 y | | | | | | | | | | | |
| 4 | randomised trials | serious ^e^ | not serious | not serious | very serious ^c,d,f^ | none | 34/352 (9.7%) | 57/372 (15.3%) | **RR 0.68** (0.48 to 0.98) | **49 fewer per 1 000** (from 3 fewer to 80 fewer) | ⨁◯◯◯ VERY LOW |
| Eczema PERIOD PREVALENCE ITT - Eczema at 2 y | | | | | | | | | | | |
| 3 | randomised trials | not serious | not serious | not serious | very serious ^b,f^ | none | 12/118 (10.2%) | 17/136 (12.5%) | **RR 0.82** (0.40 to 1.67) | **23 fewer per 1 000** (from 75 fewer to 84 more) | ⨁⨁◯◯ LOW |
| Eczema PERIOD PREVALENCE ITT - Eczema at 3 y | | | | | | | | | | | |
| 2 | randomised trials | not serious | serious ^a^ | not serious | very serious ^b,f^ | none | 7/93 (7.5%) | 9/95 (9.5%) | **RR 1.30** (0.07 to 22.67) | **28 more per 1 000** (from 88 fewer to 1 000 more) | ⨁◯◯◯ VERY LOW |
| Eczema PERIOD PREVALENCE ITT - Eczema at approx. 6-7 y | | | | | | | | | | | |
| 1 | randomised trials | not serious | not serious | not serious | very serious ^b,d^ | none | 56/167 (33.5%) | 51/162 (31.5%) | **RR 1.07** (0.78 to 1.45) | **22 more per 1 000** (from 69 fewer to 142 more) | ⨁⨁◯◯ LOW |

**CI:** Confidence interval; **RR:** Risk ratio

a. Substantial heterogeneity (I2)

b. 95% CI overlaps no effect

c. 95% CI close to no effect

d. Only one study included

e. Lack of blinding

f. Few events

**REFERENCES**

1. Chan YH, Shek LPC, Aw M, Quak SH, Lee BW. Use of hypoallergenic formula in the prevention of atopic disease among Asian children. J Paediatr Child Health 2002;38:84-88. [↑](#endnote-ref-2)
2. De Seta L, Siani P, Cirillo G, Di Gruttola M, Cimaduomo L, Coletta S. Prevention of allergic disease by an hypoallergenic formula: preliminary results at the 24 months follow-up. Med and Surgical Pediatrics 1994;16:251-4. [↑](#endnote-ref-3)
3. Lowe AJ, Hosking CS, Bennett CM, Allen KJ, Axelrad C, Carlin JB, Abramson MJ, Dharmage SC, Hill DJ. Effect of a partially hydrolyzed whey infant formula at weaning on risk of allergic disease in high-risk children: a randomized controlled trial. J Allergy Clin Immunol 2011;128:360-365.e4. [↑](#endnote-ref-4)
4. Marini A, Agosti M, Motta G, Mosca F. Effects of a dietary and environmental prevention program on incidence of allergic symptoms in high atopic risk infants: three years follow-up Acta Paediatr 1996,85:(suppl.414):1-21. [↑](#endnote-ref-5)
5. Tsai YT, Chou CC, Hsieh KH. The effect of hypoallergenic formula on the occurrence of allergic diseases in high risk infants. Zhonghua Min Guo Xiao Er Ke Yi Xue Hui Za Zhi 1991;32:137-44. [↑](#endnote-ref-6)
6. Vandenplas Y, Hauser B, Van den Borre C, Sacre L, Dab I. Effect of a whey hydrolysate prophylaxis of atopic disease. Ann Allergy 1992;68:419-24 [↑](#endnote-ref-7)
7. Vandenplas Y, Hauser B, Van den Borre C, Clybouw C, Mahler T, Hachimi-Idrissi S, et al. The long-term effect of a partial whey hydrolysate formula on the prophylaxis of atopic disease. Eur J Pediatr 1995;154:488-94. [↑](#endnote-ref-8)
8. von Berg A, Koletzko S, Grübl A, Filipiak-Pittroff B, Wichmann HE, Bauer CP, et al.; German Infant Nutritional Intervention Study Group. The effect of hydrolyzed cow's milk formula for allergy prevention in the first year of life: the German Infant Nutritional Intervention Study, a randomized double-blind trial. J Allergy Clin Immunol 2003;111:533-40. [↑](#endnote-ref-9)
9. von Berg A, Koletzko S, Filipiak-Pittroff B, Laubereau B, Grübl A, Wichmann HE, et al.; German Infant Nutritional Intervention Study Group. Certain hydrolyzed formulas reduce the incidence of atopic dermatitis but not that of asthma: three-year results of the German Infant Nutritional Intervention Study. J Allergy Clin Immunol 2007;119:718-25. [↑](#endnote-ref-10)
10. von Berg A, Filipiak-Pittroff B, Krämer U, Link E, Bollrath C, Brockow I, et al.; GINI plus study group. Preventive effect of hydrolyzed infant formulas persists until age 6 years: long-term results from the German Infant Nutritional Intervention Study (GINI). J Allergy Clin Immunol 2008;121:1442-7. [↑](#endnote-ref-11)
11. von Berg A, Filipiak-Pittroff B, Krämer U, Hoffmann B, Link E, Beckmann C, et al.; GINIplus study group. Allergies in high-risk schoolchildren after early intervention with cow's milk protein hydrolysates: 10-year results from the German Infant Nutritional Intervention (GINI) study. J Allergy Clin Immunol 2013;131:1565-73. [↑](#endnote-ref-12)
12. von Berg A, Filipiak-Pittroff B, Schulz H, Hoffmann U, Link E, Sußmann M, et al.; GINIplus study group. Allergic manifestation 15 years after early intervention with hydrolyzed formulas--the GINI Study. Allergy 2016;71:210-9. [↑](#endnote-ref-13)
13. Willems R, Duchateau J, Magrez P, Denis R, Casimir G. Influence of hypoallergenic milk formula on the incidence of early allergic manifestations in infants predisposed to atopic diseases. Ann Allergy 1993;71:147-50. [↑](#endnote-ref-14)
14. Akimoto K, Saito H, Akasawa A, Iikura Y. Preventative effect of a whey hydrolyzed formula (Nestlé, NAN H.A.) on the development of allergic symptoms in infants Arerugi 1997;46:1044-51. [↑](#endnote-ref-15)
15. Barberi I, Salpietro DC, Catalioto G, Fulia F. Clinical trial of a new hypoallergenic formula in risk infants. 2nd World Congress of Perinatal Medicine, Rome and Florence, 19-24 September 1993. [↑](#endnote-ref-16)
16. Bardare M, Vaccari A, Allievi E, Brunelli L, Coco F, de Gaspari GC, Flauto U. Influence of dietary manipulation on incidence of atopic disease in infants at risk. Ann Allergy 1993;71:366-71. [↑](#endnote-ref-17)
17. Becker A, Watson W, Ferguson A, Dimich-Ward H, Chan-Yeung M. The Canadian asthma primary prevention study: outcomes at 2 years of age. J Allergy Clin Immunol 2004;113:650-6. [↑](#endnote-ref-18)
18. Boyle RJ, Ierodiakonou D, Khan T, Chivinge J, Robinson Z, Geoghegan N, Jarrold K, Afxentiou T, Reeves T, Cunha S, Trivella M, Garcia-Larsen V, Leonardi-Bee J. Hydrolysed formula and risk of allergic or autoimmune disease: systematic review and meta-analysis. BMJ 2016;352:i974. [↑](#endnote-ref-19)
19. Chandra RK, Singh G, Shridhara B. Effect of feeding whey hydrolysate, soy and conventional cow milk formulas on incidence of atopic disease in high risk infants. Ann Allergy 1989;63:102-6. [↑](#endnote-ref-20)
20. Chandra RK, Hamed A. Cumulative incidence of atopic disorders in high risk infants fed whey hydrolysate, soy, and conventional cow milk formulas. Ann Allergy 1991;67(2Pt1):129-32. [↑](#endnote-ref-21)
21. Chandra RK, Hamed A, Prasad C, Singh GK. Culumative incidence of allergic disorders in high-risk infants fed whey hydrolysate, soy, and conventional cow’s milk formulas. Am J Clin Nutr 1992;56:758. [↑](#endnote-ref-22)
22. Chandra RK. Five-year follow-up of high-risk infants with family history of allergy who were exclusively breast-fed or fed partial whey hydrolysate, soy, and conventional cow's milk formulas. J Pediatr Gastroenterol Nutr 1997;24:380-8. [↑](#endnote-ref-23)
23. Chan-Yeung M, Manfreda J, Dimich-Ward H, Ferguson A, Watson W, Becker A. A randomized controlled study on the effectiveness of a multifaceted intervention program in the primary prevention of asthma in high-risk infants. Arch Pediatr Adolesc Med. 2000;154:657-63. [↑](#endnote-ref-24)
24. Chirico G, Gasparoni A, Ciardelli L, De Amici M, Colombo A, Rondini G. Immunogenicity and antigenicity of a partially hydrolyzed cow's milk infant formula. Allergy 1997;52:82-8. [↑](#endnote-ref-25)
25. D'Agata A, Betta P, Sciacca P, Morano C, Praticò G, Curreri R, Quattrocchi O, Sciacca F. Role of dietary prevention in newborns at risk for atopy. Results of a follow-up study. Pediatr Med Chir 1996;18:469-72. [↑](#endnote-ref-26)
26. Exl BM, Deland U, Wall M, Preysch U, Secretin MC, Shmerling DH.Zug-Frauenfeld nutritional survey (“Zuff study”): allergen-reduced nutrition in a normal infant population and its health-related effects: results at the age of six months. Nutrition Res 1998;18:1443-62. [↑](#endnote-ref-27)
27. Exl BM, Deland U, Secretin MC, Preysch U, Wall M, Shmerling DH. Improved general health status in an unselected infant population following an allergen reduced dietary intervention programme. The ZUFF-study-programme. Part I: Study design and 6-month nutritional behaviour. Eur J Nutr 2000;39:89-102. [↑](#endnote-ref-28)
28. Exl BM, Deland U, Secretin MC, Preysch U, Wall M, Shmerling DH. Improved general health status in an unselected infant population following an allergen-reduced dietary intervention programme: the ZUFF-STUDY-PROGRAMME. Part II: infant growth and health status to age 6 months. ZUg-FrauenFeld. Eur J Nutr 2000;39:145-56. [↑](#endnote-ref-29)
29. Halken S, Hansen KS, Jacobsen HP, Estmann A, Faelling AE, Hansen LG, et al. Comparison of a partially hydrolyzed infant formula with two extensively hydrolyzed formulas for allergy prevention: a prospective, randomized study. Pediatr Allergy Immunol 2000;11:149-61. [↑](#endnote-ref-30)
30. Hartman CT, Fredericks GL, Katz ES, Brown CA. Prevalence of symptomatic formula intolerance and allergy in a general infant population. J Allergy Clin Immunol 1994:210. [↑](#endnote-ref-31)
31. Iikura Y, Akimoto K, Ebisawa M, Onda T, Akazawa A, Saito H, Kimura T, Ishizawa K, Koya N. Effect of hydrolyzed whey protein formula for babies on development of allergic symptoms during infancy. Nestlé Nutrition Workshop Series "Intestinal Immunology and Food Allergy". AL de Weck & HA Sampson Eds. Nestec Ltd., Vevey / Raven Press Ltd., New York 1995;34:231-48. [↑](#endnote-ref-32)
32. Lam BCC, Yeung CY. The effect of breast milk, infant formula and hypoallergenic formula on incidence of atopic manifestation in high risk infants. Unpublished, 1992. [↑](#endnote-ref-33)
33. Laforgia N, Capolupo I, Grassi A, Latorre G, Petrillo F, Di Bitonto G, Mautone A. Atopic manifestations, growth and nutritional data in newborns at high risk for allergy fed serum protein hydrolysate: 2-year follow-up]. Pediatr Med Chir 1996;18:477-80. [↑](#endnote-ref-34)
34. Mautone A, Manzionna MM, Petrillo F, De Simone B, Di Bitonto G, Mattia MA, Clarizio L, De Mattia D. Usage of hypoallergenic formulae in the prevention of food allergy in newborns at risk. Schweiz Med Wochenschr 1991;suppl 40/II Abstract P2200*/.* [↑](#endnote-ref-35)
35. Macagno F. Utilization of a new hypoallergenic formula for feeding infants at high risk to develop cow milk protein allergy. Presented to Italian Congress of Paediatrics, Montecatini, October 1989. [↑](#endnote-ref-36)
36. Marini A, Agosti M, Motta G. A dietary prevention program including whey hydrolyzed formula for high risk atopic babies: 0-24 months follow-up. Dev Physiopathol Clin 1990;1:131-41. [↑](#endnote-ref-37)
37. Marini A, Agosti M, Motta G, et al. Prevenzione dietetic in neonati ad alto rischio atopico: follow-up 0-36 mesi: valutazioni cliniche e di laboratorio. Riv Ital Pediatr 1990;16:391-8. [↑](#endnote-ref-38)
38. Marini A, Agosti M, Motta G, Lusardi C. Dietary prophylaxis in infants with high risk of atopic diseases. 0-36 months follow-up: clinical evaluation and laboratory tests. Congress of the Italian Pediatrics Association, Cagliari. October 3-6, 1990. [↑](#endnote-ref-39)
39. Martinez Valverde A, Aljama Garcia JM. Study and validity of total and specific IgE to α-lactalbumin, β-lactoglobulin and casein, with hypoallergenic formulas and other types of nourishment. Thesis, 1993. [↑](#endnote-ref-40)
40. Nentwich I, Michkova E, Nevoral J, Urbanek R, Szepfalusi Z. Cow’s milk-specific cellular and humoral immune responses and atopy skin symptoms in infants from atopic families fed a partially (pHF) or extensively (eHF) hydrolyzed infant formula. Allergy 2001;56:1144–56. [↑](#endnote-ref-41)
41. Oldaeus G, Anjou K, Björkstén B, Moran JR, Kjellman NI. Extensively and partially hydrolysed infant formulas for allergy prophylaxis. Arch Dis Child 1997;77(1):4-10. [↑](#endnote-ref-42)
42. Porch MC, Shahane A, Elson RC, Leiva L, Sorensen RU. Effect of Early Feeding of Soy (SF), Extensively-Hydrolyzed Casein (EHCF), And Partially-Hydrolyzed Whey (PHWF) Formula on the Development of Allergic Manifestations in Children During the Second Year of Life Presentation at the American Academy of Allergy, Asthma & Immunology, New Orleans 1996 (Abstract N° 00148). [↑](#endnote-ref-43)
43. Porch MC, Shahane A, Leiva L, Elston RC, Sorensen RU. Influence of breast milk, soy or two hydrolyzed formulas on the development of allergic manifestations in infants at risk. Nutr Res 1998;18:1413-24. [↑](#endnote-ref-44)
44. Schmidt E, Eden-Kohler J, Tonkaboni F, Tolle J. Alimentary allergy prevention in infants with increased allergic risk: the effect of different feeding regimens in the first 6 months on atopic manifestations during the first year of life. A large scale feeding trial. Nestle Nutritional Workshop: Intestinal Immunology and Food Allergy. Vol. 34, Raven Press, 1995:231-48. [↑](#endnote-ref-45)
45. Schmitz J, Digeon B, Chastang C, Dupouy D, Leroux B, Robillard P, Strobel S.Effects of brief early exposure to partially hydrolyzed and whole cow milk proteins. J Pediatr 1992;121:S85-9. [↑](#endnote-ref-46)
46. Shao J, Sheng J, Dong W, Li YZ, Yu SC. Effects of feeding intervention on development of eczema in atopy high-risk infants: an 18-month follow-up study. Zhonghua Er Ke Za Zhi. 2006;44(9):684-7. [↑](#endnote-ref-47)
47. Silva Rey AL, Garcia G, Nogales A. Preventive effect of a partially hydrolysed cow milk infant formula: clinical and biological 24 month-follow up study. Thesis, 1996. [↑](#endnote-ref-48)
48. Tsai YT, Chou CC, Hsieh KH. Comparison of the occurrence of allergic diseases and milk IgE antibodies between infants fed hypoallergenic formula and those fed regular formula Nestec file, 1991. [↑](#endnote-ref-49)
49. Vaarala O, Ilonen J, Ruohtula T, Pesola J, Virtanen SM, Härkönen T, Koski M,Kallioinen H, Tossavainen O, Poussa T, Järvenpää AL, Komulainen J, Lounamaa R,Akerblom HK, Knip M. Removal of Bovine Insulin From Cow's Milk Formula and Early Initiation of Beta-Cell Autoimmunity in the FINDIA Pilot Study. Arch Pediatr Adolesc Med 2012;166(7):608-14. [↑](#endnote-ref-50)
50. Vandenplas Y, Deneyer M, Sacre L, Loeb H. Preliminary data on a field study with a new hypo-allergic formula. Eur J Pediatr 1988;148:274-7. [↑](#endnote-ref-51)
51. Vandenplas Y, Malfroot A, Dab I. Short-term prevention of cow’s milk protein allergy in infants. Immunology & allergy Practice 1989;11:430-7. [↑](#endnote-ref-52)
52. Vandenplas Y. Atopy at 3 years in high-risk infants fed whey hydrolysate or conventional formula. Lancet 1992;339:1118. [↑](#endnote-ref-53)
53. Vassella C, Buhlmann U, Battig M, Gugler E, Stadler B, Kraemer R. Interrelationship between early natural allergen challenge, nutrition, infectious disease and development of allergy: a prospective study with infants of increased allergy risk. Internal report, 1994 Abstract of presentation in Paediatric Week, Holland 1994. [↑](#endnote-ref-54)
54. Wen S, Zhijian W, Mei Z, Qiao X, Yucong Ch, Meilian D, Hongwu Ch, Ping H, Xiulan W, Meizhen T, Qianjun L, Qiwei L, Yunxia L, Chenguang X, Huiqing Ch, Zengyou L, Wei W, Yongping Y, Ruichun L, Jingran H, Ping L, Dunjin Ch. Prenatal Education and Partially Hydrolyzed Whey Formula on Infantile Allergy: a Prospective Study. Chinese Journal of Perinatology Medicine 2015;12:904-9. [↑](#endnote-ref-55)
